# Differential expression of the inflammation marker IL12p40 in the at-risk mental state for psychosis: a predictor of transition to psychotic disorder?

**DOI:** 10.1186/s12888-016-1039-7

**Published:** 2016-09-20

**Authors:** Melanie Föcking, Patrick Dicker, Lorna M. Lopez, Mary Cannon, Miriam R. Schäfer, Patrick D. McGorry, Stefan Smesny, David R. Cotter, G. Paul Amminger

**Affiliations:** 1 Department of Psychiatry, Royal College of Surgeons in Ireland, Education and Research Centre, Beaumont Hospital, Dublin 9, Ireland; 2 Department of Epidemiology and Public Health, Royal College of Surgeons in Ireland, Dublin, Ireland; 3 Orygen, The National Centre of Excellence in Youth Mental Health, The University of Melbourne Centre for Youth Mental Health and Melbourne Health, Parkville, VIC Australia; 4 Department of Psychiatry, University Hospital Jena, Jena, Germany; 5 Department of Psychiatry, Beaumont Hospital, Dublin, Ireland

**Keywords:** Plasma, At-risk mental state, IL12/IL23p40, Omega-3, Inflammation, Biomarker, Psychosis

## Abstract

**Background:**

The identification of biomarkers of transition from the at-risk mental state (ARMS) to psychotic disorder is important because early treatment of psychosis is associated with improved outcome. Increasing evidence points to an inflammatory contribution to psychosis.

We questioned whether raised levels of plasma inflammatory markers predict transition from ARMS to psychotic disorder and whether any such predictors could be reduced by omega-3 (ω-3) polyunsaturated fatty acids (PUFAs).

**Methods:**

We measured the levels of 40 neuroinflammation biomarkers using a commercially available immunoassay kit. Firstly, we compared inflammatory markers in subjects in the ARMS who transitioned to psychotic disorder (*n* = 11) compared to subjects who did not (*n* = 28). Then we compared inflammatory markers in all subjects before and after ω-3 PUFA treatment (*n* = 40).

**Results:**

Our data provides preliminary evidence that elevations in the baseline plasma levels of the inflammatory marker IL12/IL23p40 are associated with transition from ARMS to psychotic disorder. IL12/IL23p40 levels did not change following 12 weeks administration of ω-3 PUFAs. These findings provide evidence that elevated plasma IL12/IL23p40 is a potential biomarker of increased risk for transition to psychotic disorder.

**Conclusion:**

Further studies are required to confirm and extend this finding. Our results do not provide support for the possibility that administration of ω-3 PUFAs act to reduced transition to psychotic disorder by reducing blood levels of IL12/IL23p40.

**Trial registration:**

ClinicalTrials.gov, a service of the U.S. National Institutes of Health, Identifier: NCT00396643, last updated December 20, 2007. Retrospectively registered.

## Background

Over the past few years there has been a substantial interest in the role of inflammation in “priming” the brain for psychosis based on epidemiological, post-mortem, genetic and therapeutic data [1, 2]. Elevated levels of cytokines and other markers of inflammation have been reported in schizophrenia and mood disorder [3–6].

Epidemiological studies have shown that elevations in maternal C-reactive protein (CRP) and pro-inflammatory markers, such as cytokines IL-8 and TNFα, in addition to elevations in IL6 in childhood, predict psychotic disorder [1, 7, 8]. It has been proposed that exposure to an elevated inflammatory state in prenatal life or early childhood, possibly in association with a genetic vulnerability towards dysregulation of the immune system, “primes” [9] the brain and leads to vulnerability state which may be expressed clinically as early psychotic symptoms [10]. Further stressors during development such as adolescent bullying, trauma, stressful life transitions or substance use may serve to convert this vulnerability into disorder [11]. The primary cause of the elevated ‘inflammatory tone’ is not known. Maternal infection is an obvious causal candidate [12–15], but inflammation may also reflect a stress-related loss of normal glucocorticoid-associated anti-inflammatory tone [16] and represent a common pathway mediating a diverse range of pregnancy-related and early childhood exposures associated with psychotic illness [17].

Early treatment of psychosis is associated with improved outcome [18]. However there is no biomarker signature predicting those at increased risk of developing a psychotic disorder among those already known to be at risk due to their symptom profile, the so-called ‘at-risk mental state’ (ARMS) criteria for psychosis. Approximately 30–40 % of subjects in the ARMS convert to psychotic disorder [19]. While inflammatory biomarkers are clearly important in psychosis, few previous studies have examined whether blood markers of inflammation distinguish those who convert to psychotic disorder from those who do not [6, 20]. The National Institute of Mental Health has prioritised the identification of such biomarkers as targets for the development of new therapies. Among such potential therapeutic agents are omega-3 fatty acids which have been shown to protect subjects in the ARMS from developing acute psychotic disorder [21].

Amminger and colleagues recently reported on the effects of long-chain omega-3 (ω-3) polyunsaturated fatty acids (PUFAs) for indicated prevention of psychosis in adolescents and young adults at risk of developing psychosis according to ARMS criteria. The results of this trial indicated that a 12-week intervention with ω-3 PUFAs significantly reduced the risk of transition to psychosis at 1 year follow-up [21, 22]. The ω-3 PUFA group also showed greater improvement in positive, negative and general symptoms as well as in functioning. It is of interest that the protective effects were sustained for the 12-month follow-up period [22], an effect that could be explained by the neuroprotective properties of ω-3 PUFAs [23]. Given the importance of inflammation in psychotic disorder we asked whether the presence of raised markers of inflammation among ARMS subjects could distinguish those who transition to psychotic disorder (ARMS-P) from those who do not (ARMS-NP). We also asked whether ARMS subjects treated with ω-3 PUFAs showed a reduction in any of such inflammatory plasma markers.

## Methods

### Plasma samples

Plasma samples collected from subjects aged 13–25 years meeting at least one of three operationally defined criteria for increased risk for psychosis (i.e., attenuated psychotic symptoms, brief limited intermittent psychotic symptoms, or a genetic risk with decreased functioning) and well-validated groups of risk for psychosis according to [19, 24–26] were included in the study if they did not fulfill any of the exclusion criteria. These included a previous history of a psychotic disorder or manic episode, a substance-induced psychotic disorder, acute suicidal or aggressive behavior, a current substance dependence except for cannabis dependence, neurological disorder, relevant structural brain changes, IQ of less than 70, previous antipsychotic or mood-stabilizing treatment for longer than 1 week, previous supplementation with ω-3 PUFAs within 8 weeks of inclusion in the trial, abnormal laboratory values for transaminases, thyroid hormones, C-reactive protein, bleeding parameters or any other severe intercurrent illness that may have put the person at risk. For further details please see [21, 22].

### Study design

Subjects studied were included in a double-blind, placebo-controlled randomized controlled trial with a 12-week intervention period of 1.2 g/d ω-3 PUFAs or placebo (Trial registration: clinical trials.gov Identifier: NCT00396643). During the intervention period participants received weekly assessments for 4 weeks and then at 8 and 12 weeks, as well as at the 6- and 12-month follow-up [22]. All patients who progressed to a first episode of psychosis were independently confirmed by non-project psychiatrists and then treated accordingly if needed. The Structured Clinical Interview for DSM-IV-TR Axis I Disorders was used to ascertain psychiatric diagnoses at baseline, at 12-months follow-up, and at 12 months and was also supplemented by additional sources, e.g. medical records review (please see Amminger et al. [21] for further details). Eighty-one patients agreed to participate in the trial and gave informed written consent. They were then assigned to one of the two intervention arms (*n* = 41 ω-3 PUFA, *n* = 40 placebo) [22]. From these samples the majority were available for the current study (*n* = 40 ω-3 PUFA, *n* = 39 placebo). Because the conversion rates differed significantly between the treatment groups in our treatment trial, and to eliminate treatment effects, we only investigated those participants who received placebo in the prediction analysis. The study was approved by the Medical University of Vienna ethics committee, and written informed consent was obtained from all participants (parental or guardian consent was obtained for those aged under 18 years). The study was also approved by the local ethics committee (Royal College of Surgeons in Ireland Research Ethics Committee (Application No. REC 766)).

### Assessment of inflammation markers - Immunological assays

Neuroinflammatory biomarkers were measured with the V-PLEX Human Biomarker 40-Plex Kit (Mesoscale Discovery (MSD), Maryland, US) according to the manufacturer’s instructions. In brief, 250 μl plasma (10–50 μl for each of the five plates) was diluted as recommended in buffer and 25 μl applied to the bottom of each well in duplicate, incubated for two hours, washed three times and a solution containing detection antibodies was applied and incubated for another two hours. Subsequently the plate was washed again three times before the read buffer was applied and the plate read in an MSD instrument. The instrument measures the intensity of emitted light to provide a quantitative measure of analytes in the sample.

Forty markers were quantitatively measured at the same time. This allowed simultaneous detection of cytokines and chemokines with high precision and accuracy. These analytes were: bFGF, CRP, Eotaxin, Eotaxin-3, Flt-1, GM-CSF, ICAM-1, IFN-γ, IL-1α, IL-1β, IL-10, IL-12 p70, IL-12/IL-23p40, IL-13, IL-15, IL-16, IL-17A, IL-2, IL-4, IL-5, IL-6, IL-7, IL-8, IL-8 (high abundance), IP-10, MCP-1, MCP-4, MDC, MIP-1α, MIP-1β, PlGF, SAA, TARC, Tie-2, TNF-α, TNF-β, VCAM-1, VEGF, VEGF-C, VEGF-D.

Each sample was tested in duplicate and the levels reported represent the mean of the duplicates. Sensitivity and coefficient of variation measures were within their expected ranges.

### Statistical analysis

For statistical analysis, calculated concentration levels were first checked to be within detection limits of the assay and having a coefficient of variation of at most 20 %. The data were log transformed to remove skewness or the potential influence of outliers.

In the placebo group differences between those ARMS-P (*n* = 11) and ARMS-NP (*n* = 28) were determined using a two-sample t-test and a non-parametric analysis (Wilcoxon rank-sum test). Potential confounders, namely body mass index, age, gender, tobacco use, alcohol use, exposure to illicit drugs, marijuana use, antidepressant medications and benzodiazepine use were compared between groups. Where significant differences between these confounding factors were identified (see Table 1) these were included as co-variates in a secondary analysis.

**Table 1:** Demographic information of study subjects. Data are expressed as mean +/- s.e.m.

	Placebo			Omega3
	ARMS-NP	ARMS-P	*p*-value	
N	28	11		40
Age Years (SD)	16.24 (0.34)	15.88 (0.33)	0.545	16.9 (0.38)
Gender (female/male)	20/8	7/4	0.635	26/14
BMI (kg/m^2^)	21.17 (0.61)	21.3 (1.08)	0.906	21.05 (0.66)
Smoking (yes/no)	17/11	7/4	0.866^a^	17/23
Alcohol use (rarely/regularly)	15/13	6/5	0.956^a^	23/17
Marijuana use (yes/no)	3/25	3/8	0.197^a^	6/34
Prescription medication				
Antidepresant (yes/no)	9/19	4/7	0.801^a^	14/26
Benzodiazepine (yes/no)	1/27	2/9	0.123^a^	7/33

^a^Indicates where a chi-Square comparison was used to analyse the data

Because of the known potential importance of smoking on inflammatory cytokine markers, for any candidate inflammatory marker showing differential expression between groups, we also compared its levels between smokers and non smokers in ARMS-NP alone to determine the effect of smoking on that specific cytokine.

In order to test the effect of ω-3 PUFAs treatment we performed a repeated measures analysis of cytokine levels compared between baseline and after 12 weeks treatment (*n* = 40).

SAS Version 9.2 was used for data management and statistical analysis.

## Results

**Fig. 1:**
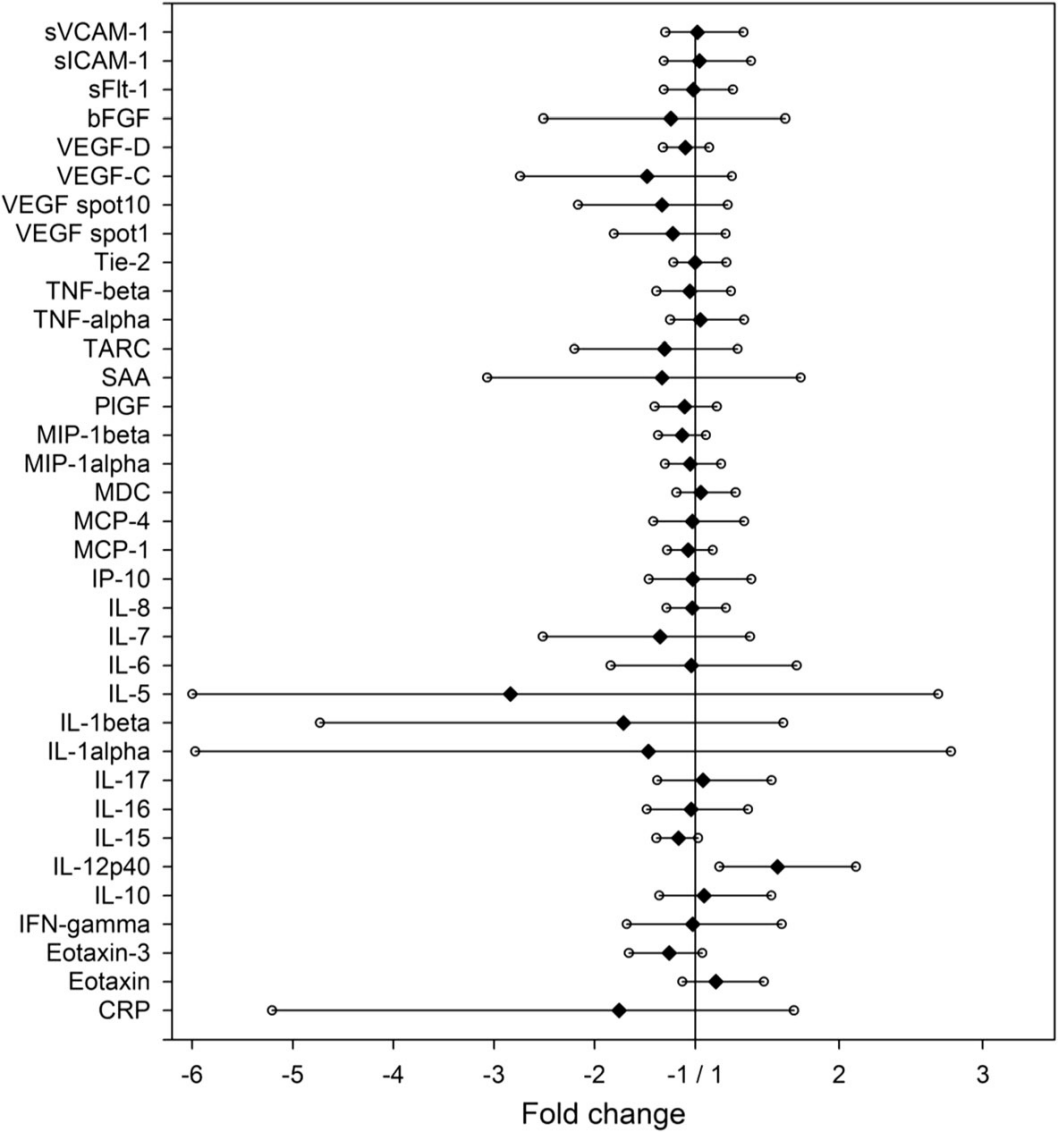
Changes in baseline inflammatory marker levels between ARMS-NP and ARMS-P. Marker fold changes and 95 % confidence interval are shown

We performed our experiments in baseline plasma samples from 39 individuals in the ARMS placebo and 40 subjects in the ω-3 PUFA treatment group (Table 1) [21]. Participants were followed up for 1 year during which time they received non-neuroleptic state-of-the-art care unless they transitioned to psychotic disorder. For a detailed description of therapeutic interventions see Amminger et al. 2010 [21]. After 1 year, 11 of 39 individuals of the placebo group transitioned to psychotic disorder and 28 individuals remained at risk (in omega-3 PUFA treated group 2/40 transitions). Fold changes for each marker between ARMS-P and ARMS-NP groups are listed in Fig. 1.

No group differences in body mass index, age, gender, tobacco use, and alcohol use, exposure to illicit drugs, marijuana use, antidepressant medications and benzodiazepine use were observed and therefore with the exception of BMI and smoking these co-variates were not included in our analysis comparing the levels of inflammatory markers between groups (Table 2 and Fig. 1).

**Table 2:** Changes in baseline inflammatory marker levels between ARMS-NP and ARMS-P. Mean values, fold changes and *p*-values are shown

Marker	ARMS-NP mean [pg/ml]	ARMS-P mean [pg/ml]	Fold change	*p*-value converter
CRP	597752	340155	-1.7573	0.2979
Eotaxin	81.22	92.825	1.1429	0.2969
Eotaxin-3	13.06	10.378	-1.2584	0.0997
IFN-γ	5.0019	4.8783	-1.0253	0.9190
IL-10	0.2562	0.2717	1.0607	0.7452
IL-12p40/IL23	98.095	154.19	1.5719	0.0043
IL-15	2.1771	1.8656	-1.1669	0.0790
IL-16	169.02	162.19	-1.0421	0.8128
IL-17	2.2659	2.3853	1.0527	0.7782
IL-1α	3.3439	2.2814	-1.4657	0.5685
IL-β	0.1647	0.0962	-1.7128	0.2683
IL-5	1.5655	0.5521	-2.8355	0.2708
IL-6	0.3867	0.372	-1.0394	0.8902
IL-7	3.7935	2.8095	-1.3502	0.3314
IL-8	3.7555	3.6444	-1.0305	0.7863
IP-10	267.65	261.03	-1.0254	0.8869
MCP-1	68.28	63.834	-1.0696	0.4551
MCP-4	45.132	43.87	-1.0288	0.8587
MDC	1105.3	1147.5	1.0382	0.7187
MIP-1α	13.325	12.682	-1.0507	0.6390
MIP-1β	54.783	48.465	-1.1304	0.2071
PIGF	47.649	43.104	-1.1054	0.4018
SAA	1.24E + 06	934565	-1.3303	0.4908
TARC	100.52	77.035	-1.3048	0.3085
TNF-α	2.2792	2.3577	1.0344	0.7914
TNF-β	0.2566	0.2434	-1.0541	0.6914
Tie-2	11001	10973	-1.0025	0.9798
VEGF	86.718	70.914	-1.2229	0.3055
VEGF	31.114	23.396	-1.3299	0.2421
VEGF-C	179.68	121.52	-1.4785	0.1958
VEGF-D	851.82	775.19	-1.0988	0.3087
bFGF	25.327	20.382	-1.2426	0.5351
sFlt-1	90.022	88.16	-1.0211	0.8671
sICAM-1	215136	221081	1.0276	0.8545
sVCAM-1	427575	433463	1.0138	0.9203

**Fig. 2:**
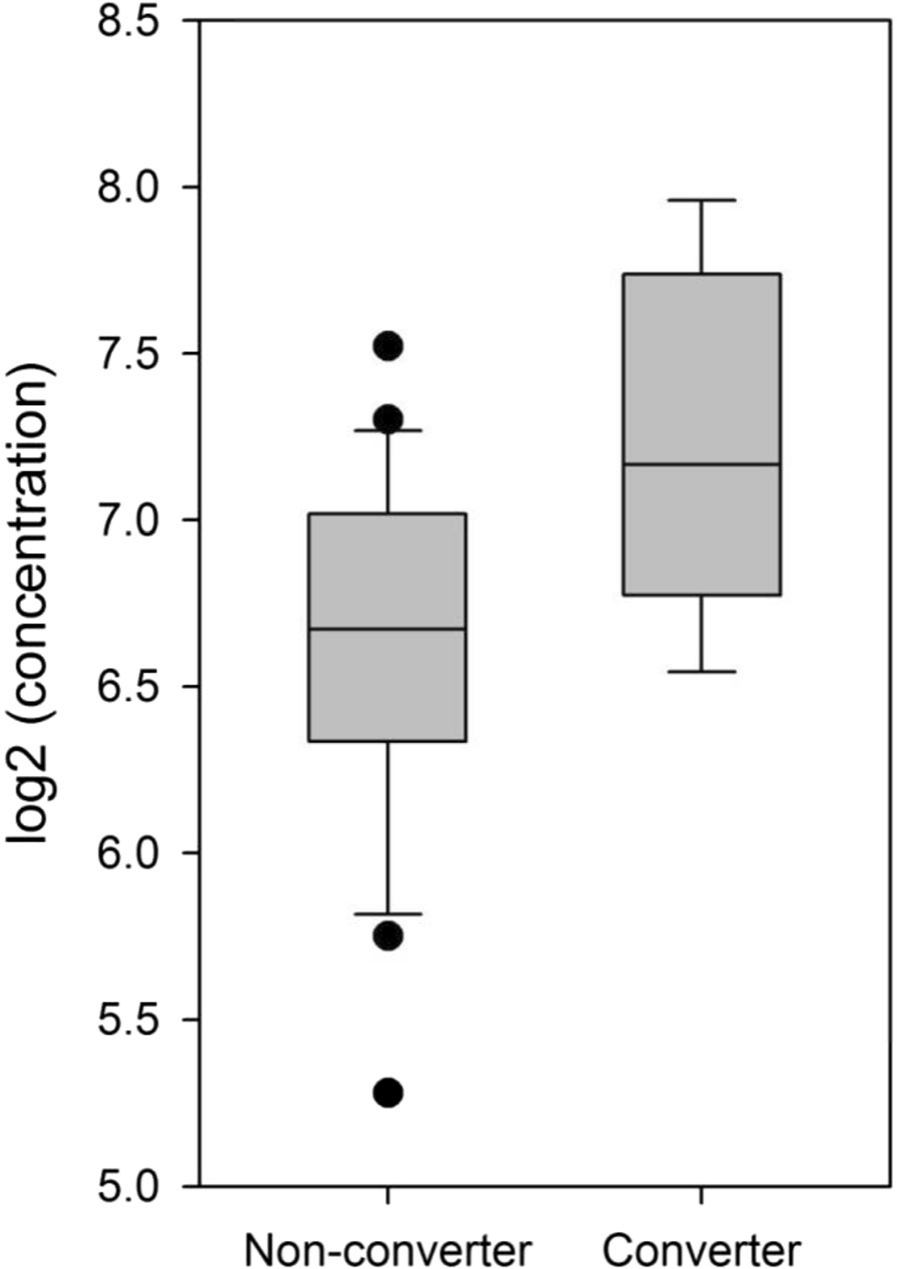
Box plots for IL12/23 in plasma from ARMS-NP and ARMS-P. Each point represents the concentration of IL12/23 (pg/ml) in a single plasma sample. Horizontal lines show median values for each group

Of the 40 biomarkers that were assayed, 35 passed quality control (CRP, Eotaxin, Eotaxin 3, ICAM-1, IFNγ, IL-10, IL-12/IL23p40, IL-15, IL-16, IL-17, IL-1α, IL-1β, IL-5, IL-6, IL-7, IL-8, IP-10, MCP-1, MCP-4, MDC, MIP-1α, MIP-1β, PIGF, SAA, sFLT-1, TARC, Tie-2, TNFα, TNFβ, VCAM-1, VEGF, VEGF-A, VEGF-C VEGF-D, bFGF). One marker was found to be significantly increased in ARMS-P compared with ARMS-NP – Interleukin 12/23 subunit p40 (IL12/IL23p40, henceforth called IL12/23) (1.6 fold, *p* = 0.0043, Fig. 2). This however did not survive formal Bonferroni correction (required *p*-value for 35 tests *p* < 0.0014). In addition, to account for potential confounding effects of smoking, ANCOVA including Body Mass Index (BMI) and smoking as covariates was undertaken as these variables are known from the literature to have effects on inflammatory markers (see e.g. [27, 28]). Group differences in IL12/23 changed to (1.47 fold, *p* = 0.0025) and this effect was not significantly affected by BMI or smoking. In addition, no significant differences in IL12/23 levels were observed between ARMS-NP smokers versus non smokers (1.23 fold, *p* = 0.213).

Given the evidence that IL12/23 is elevated among ARMS-P compared to ARMS-NP and the data suggesting clinical efficacy of ω-3 fatty acids in preventing transition from ARMS-NP to ARMS-P [21], we hypothesized that treatment with ω-3 PUFA will reduce levels of the inflammatory cytokine IL12/23. We investigated this question by comparing IL12/23 levels at baseline with 12 weeks plasma samples in 40 ARMS subjects who were treated with ω-3 PUFA over a 12 week period. The neuroinflammation biomarker IL12/23 passed quality control and no significant difference was observed in its levels before and after the 12 weeks ω-3 PUFA treatment (fold change -1.02, *p* = 0.82) regardless of correction for the potential confounders.

## Discussion

This study identifies the plasma inflammatory marker IL12/23 as a potential candidate biomarker of transition from the ARMS to psychotic disorder (ARMS-P). Our data is consistent with previous reports implicating inflammatory markers in psychosis [5, 29, 30] and mood disorders [3, 5] but is among the first to identify an inflammatory marker which distinguishes subjects in the ARMS who will transition to psychotic disorder at 1 year follow-up compared from those who will not. The finding is important as it may help to identify those who will benefit from earlier, more effective treatment [31].

IL-12p40 plays critical roles in regulating the inflammatory response such as that to prenatal and early developmental infections [32], and autoimmunity [33] both of which have been implicated in schizophrenia [34]. Our observations of elevated IL12/23 relates specifically to the p40 subunit of IL-12, which can form a heterodimer with p19 to form IL-23, or with the p35 subunit to form IL-12p70 [35]. In keeping with our observation of increased IL12, upregulation of IL-17 and Th17 cells has been observed in first episode drug naïve schizophrenia [36], although another study of first episode drug naïve subjects demonstrated a reduction in IL12p40 compared to matched healthy controls [29]. In keeping with our findings a meta-analysis of serum cytokine alterations in schizophrenia or related psychotic disorder patients found increased levels of interleukin, among those IL6, IL12, TNF-α, TGF-β although with significant heterogeneity [37].

Three recent studies have investigated inflammatory markers in ARMS subjects in addition to other markers of oxidative stress and metabolism and hormones [4, 6, 20]. Perkins and colleagues (2015) compared plasma samples of clinically high risk (CHR) subjects who developed psychosis with CHR who did not, and observed a set of 15 biomarkers which distinguished ARMS and schizophrenia subjects combined from healthy controls, and also provided preliminary evidence, that some inflammatory and stress related proteins distinguish ARMS from schizophrenia [6]. Hayes and colleagues (2014) studied cerebrospinal fluid from subjects in the ARMS and schizophrenia and observed another different set of 15 inflammatory markers (only IL8 overlapping with Perkins and colleagues (2015)) that distinguished ARMS and schizophrenia combined from normal controls (*n* = 35) and a subset which distinguished ARMS subjects from schizophrenia, although once again significant differences did not survive correction for multiple testing [4]. Stojanovic and colleagues (2014) studied IL6 and CRP levels in ARMS, psychotic disorder and normal controls and observed elevated IL6 in the combined ARMS and psychotic disorder groups compared to controls, and non significant elevations among ARMS who transitioned compared to those who did not [20]. Together with our own data these findings offer promise that altered levels of inflammatory cytokines and indeed hormones and stress markers may contribute usefully as a component of a risk prediction tool for transition from ARMS to psychotic disorder [38]. However, further work is required to replicate and refine the risk signature and to combine blood or CSF-based biomarkers with the most robust neuropsychological, neuroimaging and genomic risk predictors [39]. Of note, while Perkins and colleagues (2015) included IL12/23 in their panel they did not include it in group comparisons because it was below the levels of detection in >20 % of their sample [6]. Specifically in relation to inflammatory markers it will also be critical to characterise the changes longitudinally and in relation to ARMS, psychotic disorder, psychiatric disorder generally, severity of illness, and potentially to administered drug treatments. Furthermore, the possibility that there are distinct inflammatory subtypes of psychosis must be considered. A mechanistic role of IL12 in psychosis formation could be microglia activation [40] and postmortem studies suggest that there is an inflammatory subgroup of schizophrenia with pronounced microglial activation [41].

Having identified IL12/23 as a potential biomarker of transition from ARMS to psychotic disorder, we tested whether treatment of ARMS subjects with ω-3 PUFA over a 12 weeks period reduced the levels of IL12/23 when comparing baseline and week 12 plasma samples. No such reduction was observed. This suggests that ω-3 PUFA do not act through reductions in IL12/23 when mediating their proposed antipsychotic [42] or psychosis preventing effects [43]. Previous studies have suggested that ω-3 PUFA may have anti-inflammatory effects which mediate their clinical efficacy in depression [44], neurodegenerative disorders [45] and psychosis [21, 46] although to our knowledge no previous study has assessed the effects of ω-3 PUFA augmentation on inflammatory cytokine levels in the ARMS. While our findings provide no support for the view that ω-3 PUFA may act by reducing candidate inflammatory cytokines, further work is required as ω-3 PUFA may require longer treatment periods or different doses to exert effects on inflammatory cytokines, or may be only effective in an inflammatory subgroup of at risk subjects.

Our study has some weaknesses that need to be considered. Firstly, the sample may be viewed as relatively small. Our study involved 39 ARMS subjects in the placebo group (of whom 11 transitioned to psychosis at 1 year follow-up) and 40 in the ω-3 PUFA treatment group. While these numbers limit the statistical power to detect small differences between groups it should be appreciated that these are unique samples that require a great investment of resources to identify and follow-up. Secondly, normal controls were not available and as a result we could not characterise whether ARMS subjects generally demonstrate elevations in inflammatory markers compared to normal controls as suggested by previous studies in ARMS groups [4, 6]. Thirdly, the study used the same inclusion and exclusion criteria originally proposed by the PACE Clinic in Melbourne [47]. In these studies cannabis dependency was not excluded because this would have resulted in a non-representative Ultra-High-Risk sample, since the rates for cannabis dependency were very high in the Ultra-High-Risk patients. However, as in the main outcome papers from the cohort used in this work [21, 48], the rates at baseline were quite low for substance abuse in general as well as for cannabis. Fourthly, our study did not assess the broad array of markers involving oxidative stress and metabolic function that have been implicated by other studies of post-mortem brain [41, 49] and plasma in ARMS. More work is needed to target these markers. Fifthly, the issue of potential confounders and multiple comparisons need to be considered. Our finding of increased IL12/23 in ARMS-P compared to ARMS-NP while highly significant did not survive formal correction for multiple comparisons according to Bonferroni adjustment and thus must be viewed as preliminary and in need of further replication. Nonetheless IL12/23 has been implicated previously in psychosis and our findings support and extend these latter findings [29]. BMI and smoking can also influence plasma cytokine levels and inclusion of these variables in our ANCOVA slightly reduced the significance of the findings. Such a correction may not however have been appropriate as IL12/23 did not differ among ARMS-NP who smoked compared to those who did not, and BMI was not different between groups. Finally, outcome data in terms of transition to psychotic disorder is based on 1 year follow-up and therefore our findings are only relevant in terms of this relatively short-term risk of transition to psychotic disorder. It is possible that other inflammatory markers may predict longer term outcome. As longer term outcome data becomes available we plan to reanalyse the data to clarify if different markers predict longer term outcome.

## Conclusions

In conclusion, our data indicates that an elevation in the plasma level of the inflammatory cytokine IL12/23 may distinguish subjects in the ARMS who will develop a psychotic disorder from those who will not. Future work is needed to confirm this finding in larger cohorts and to characterise the longitudinal pattern of changes in inflammatory markers over time and in relation to illness staging.

## Abbreviations

ANCOVA: Analysis of covariance
ARMS: At-risk mental state
ARMS-NP: At-risk mental state- non psychotic
ARMS-P: At-risk mental state- psychotic
BMI: Body mass index
PUFA: Poly-unsaturated fatty acids
ω-3: Omega-3
